# Effects of spatial smoothing on functional brain networks

**DOI:** 10.1111/ejn.13717

**Published:** 2017-10-20

**Authors:** Tuomas Alakörkkö, Heini Saarimäki, Enrico Glerean, Jari Saramäki, Onerva Korhonen

**Affiliations:** ^1^ Department of Computer Science School of Science Aalto University PO Box 15400 FI‐00076 Aalto Espoo Finland; ^2^ Department of Neuroscience and Biomedical Engineering School of Science Aalto University Espoo Finland; ^3^ Turku PET Centre University of Turku Turku Finland

**Keywords:** centrality, connectomics, functional magnetic resonance imaging, network hubs, preprocessing

## Abstract

Graph‐theoretical methods have rapidly become a standard tool in studies of the structure and function of the human brain. Whereas the structural connectome can be fairly straightforwardly mapped onto a complex network, there are more degrees of freedom in constructing networks that represent functional connections between brain areas. For functional magnetic resonance imaging (fMRI) data, such networks are typically built by aggregating the blood‐oxygen‐level dependent signal time series of voxels into larger entities (such as Regions of Interest in some brain atlas) and determining their connection strengths from some measure of time‐series correlations. Although it is evident that the outcome must be affected by how the voxel‐level time series are treated at the preprocessing stage, there is a lack of systematic studies of the effects of preprocessing on network structure. Here, we focus on the effects of *spatial smoothing*, a standard preprocessing method for fMRI. We apply various levels of spatial smoothing to resting‐state fMRI data and measure the changes induced in functional networks. We show that the level of spatial smoothing clearly affects the degrees and other centrality measures of functional network nodes; these changes are non‐uniform, systematic, and depend on the geometry of the brain. The composition of the largest connected network component is also affected in a way that artificially increases the similarity of the networks of different subjects. Our conclusion is that wherever possible, spatial smoothing should be avoided when preprocessing fMRI data for network analysis.

## Introduction

It is broadly accepted in the neuroscience community that the human brain consists of interconnected, functionally specialized areas. Thus, the brain can be naturally modeled as a complex network (Wig *et al*., 2011; Sporns, 2013a,b). In the network approach, nodes of the network represent brain areas, and links depict their structural or functional connections. The structural features of the network may then help to understand the function of the brain.

Network analysis of functional magnetic resonance imaging (fMRI) data has revealed that the brain has a non‐random, hierarchical core‐periphery structure; network studies have also helped to identify hubs, that is, central areas of the brain. Functional brain networks have been reported to change with age, between health and disease, and between different cognitive tasks. For reviews, see Papo *et al*. (2014), Bassett & Bullmore (2009), and Sporns (2013b).

It has been criticized that in fMRI studies in general, the choice of analysis parameters is often justified insufficiently and reported incompletely (Carp, 2012). This includes functional brain network studies. There are fundamental gaps in understanding: the effects of different data acquisition and preprocessing methods on the structure of functional networks are not well understood. Indeed, factors that affect the reliability of the fMRI network studies have lately become a subject of discussion (Shehzad *et al*., 2009; Telesford *et al*., 2010; Braun *et al*., 2012; Hayasaka, 2013; Andellini *et al*., 2015; Aurich *et al*., 2015; Shirer *et al*., 2015).

In this study, we concentrate on *spatial smoothing*, a commonly applied preprocessing method that may affect network properties (Fornito *et al*., 2013; Stanley *et al*., 2013). In the smoothing process, the signal from each measurement voxel is redefined as the average of the signals of the voxel itself and its neighbors; typically, a smoothing kernel is applied when averaging. Spatial smoothing belongs to the standard set of fMRI preprocessing methods when the general linear model (GLM) is used as the analysis paradigm: smoothing by a Gaussian kernel ensures that data fulfill the Gaussianity assumption of the model (Mikl *et al*., 2008). Spatial smoothing also increases the signal‐to‐noise ratio (SNR), compensates for inaccuracies in spatial registration, and decreases inter‐subject variability (Hopfinger *et al*., 2000; Triantafyllou *et al*., 2006; Mikl *et al*., 2008; Bennett & Miller, 2010; Pajula & Tohka, 2014). Spatial smoothing is often applied outside the GLM paradigm as well; in this case, the justification for using it is less evident.

In seed‐based functional connectivity studies, spatial smoothing has been reported to increase the connection strength between voxels, measured in terms of the correlation coefficient, leading to detection of larger clusters of voxels connected with the seed (Wu *et al*., 2011; Molloy *et al*., 2014). Meanwhile, smoothing decreased the differences in connectivity between different spatial resolutions (Molloy *et al*., 2014). Scheinost *et al*. (2014) found that image smoothness correlates with seed‐based connectivity and with the degree measured in networks where nodes represent measurement voxels. Although the different image smoothness values at different voxels are mostly caused by motion artefacts (Scheinost *et al*., 2014), one may expect to see similar effects if spatial smoothing applied at the preprocessing stage leads to differences in image smoothness in different parts of the brain. In the case of Regional Homogeneity that measures local connectivity, spatial smoothing decreased test–retest reliability (Zuo *et al*., 2013).

The above results indicate that spatial smoothing may affect the properties of functional brain networks. However, to the best of our knowledge, the effects of spatial smoothing on the structure and properties of region‐level functional brain networks have not been investigated in detail.

In this study, we investigate how spatial smoothing affects the structure of functional brain networks. To this end, we use two independent datasets: resting‐state fRMI data of 13 subjects measured in‐house as well as 28 subjects from the Autism Brain Imaging Data Exhange I (ABIDE I) initiative. For each of these subjects, we construct the resting‐state functional network using anatomically defined Regions of Interest (ROIs) as network nodes. We investigate the effects of spatial smoothing on several aspects of network structure, including the distribution of link lengths, the identity of the most central *hubs* of the network, and the structure of the largest connected component (LCC) that forms the core of the network.

## Methods

### Subjects

The in‐house data used in this study are from 13 healthy, right‐handed subjects (11 females, 2 males, age 25.1 ± 3.9, mean ± SD). They all had normal or corrected‐to‐normal vision, and none of them reported a history of neurological or psychiatric disease. All subjects volunteered for the study and gave a written, informed consent according to the Declaration of Helsinki. Subjects were compensated for their participation. This study was approved by the Research Ethics Committee of Aalto University.

### Data acquisition

Functional magnetic resonance imaging data were acquired with a 3T Siemens Magnetom Skyra scanner in the AMI Centre (Aalto Neuroimaging, Aalto University, Espoo, Finland). A whole‐brain T2*‐weighted EPI sequence was collected with the following parameters: TR = 1.7 s, 33 axial slices, TE = 24 ms, flip angle = 70, voxel size = 3.1 × 3.1 × 4.0 mm, matrix size 64 × 64 × 33, FOV 198.4 × 198.4 mm. Data from an approximately 6 min (215 time points) resting‐state session were used in this study. In the resting‐state condition, subjects were instructed to lay still with their eyes open, gaze fixated to a gray background image, and avoid falling asleep.

Structural MR images with isotropic 1 × 1 × 1 mm voxel size were acquired using a T1‐weighted MP‐RAGE sequence.

### Preprocessing of the fMRI data

For preprocessing, we used FSL (Smith *et al*., 2004; Woolrich *et al*., 2004; Jenkinson *et al*., 2012) and an in‐house MATLAB toolbox, BraMiLa (https://version.aalto.fi/gitlab/BML/bramila). First, the three‐first frames of each subject's data were removed to minimize the error caused by the scanner transient effect. This left a time series of 212 time points for further analysis. The preprocessing pipeline continued with slice timing correction, motion correction by MCFLIRT (Jenkinson *et al*., 2002), and extraction of white matter and cerebrospinal fluid (CSF). Functional data were co‐registered to the anatomical image with FLIRT (7 degrees of freedom), registered to MNI152 template (12 degrees of freedom), and downsampled to voxels of 4 × 4 × 4 mm. Signals were linearly detrended, and signals from white matter and CSF were regressed out from the data.

Expansion of motion parameters was extracted from the data with linear regression (36 Volterra expansion based signals) (Power *et al*., 2014) to control for motion artifacts. As head motion is a possible source of artifacts in connectivity studies (Power *et al*., 2012), the framewise displacement was calculated for each subject, but its values were under the suggested threshold of 0.5 mm. Therefore, no scrubbing was performed.

To eliminate further artifacts, voxels that were located at the boundary of the brain and the skull with a mean signal power less than 2% of the individual's mean signal power were excluded from the analysis.

### Spatial smoothing

In spatial smoothing, the time series of each voxel is redefined as an average of the time series of neighboring voxels, weighted by a smoothing kernel:

(1) $$x_{i}=\frac{\sum_{j}G_{i}\left(j\right)x_{j}}{\sum_{j}G_{i}\left(j\right)},$$

where *x*_*i*_ denotes the time series of voxel *i*,* G*_*i*_(*j*) is the value at voxel *j* of the smoothing kernel *G*_*i*_ centered at voxel *i*, and the summation is over all voxels. For the majority of these voxels, *G*_*i*_(*j*) ≈ 0.

Spatial smoothing was always applied as the last preprocessing step before network extraction. We used three Gaussian kernels with different full width at half maximum (FWHM): 5, 8, and 12 mm. As a reference, we used non‐smoothed data (FWHM 0 mm).

The chosen kernel sizes are commonly used among the fMRI community and have been recommended in the literature. Some researchers have suggested that kernel size ‘should approximate the size of the underlying signal or evoked response’ that would be approximately 3–5 mm on the cortex (Hopfinger *et al*., 2000). Others have argued that kernel size should be 2–3 times the voxel size (Mikl *et al*., 2008; Pajula & Tohka, 2014).

### Regions of interest

We divided the cortex into 96 anatomical ROIs. The ROIs were from the HarvardOxford (HO) atlas (http://neuro.debian.net/pkgs/fsl-harvard-oxford-atlases.html) (Desikan *et al*., 2006) at 30% probability level (i.e., in the group used to create the parcellation, a voxel belongs to the ROI that it is associated with in 30% or more of the subjects). The ROIs did not overlap; each voxel belonged to one ROI only.

We deliberately chose to follow the pipelines that are commonly adopted in connectomics. These pipelines often exclude the cerebellum and subcortical areas. Therefore, these areas were not included in our analysis, despite the important role that the cerebellum and subcortical areas have in brain function (for an extensive review, see Koziol & Budding, 2009).

The ROI time series were defined as the average over the time series of the voxels in the focal ROI:

(2) $$X_{I}=\frac{1}{N_{I}}\sum_{i\in I}x_{i},$$

where *I* is the focal ROI, *N*_*I*_ is the size of the ROI *I* measured in voxels, and *x*_*i*_ is the time series of voxel *i*. The sizes of the ROIs varied between 5 and 857 with the mean ROI size being 141.58 ± 147.46 (mean ± SD). The median ROI size was 88, and the majority of the ROIs consisted of approximately 100 voxels. For details on ROI sizes, see Supporting Information Table S1.

### Network extraction

We used the ROIs as the nodes of the functional brain network of each subject. The link weights between each pair of ROIs were defined as the Pearson correlation coefficient of their time series. This resulted in a symmetrical adjacency matrix *A*, where the element *A*_*I*,*J*_ indicated the strength of correlation between ROIs *I* and *J*. We set the diagonal to *A*_*I,I*_ = 0 to exclude self‐links that contain no useful information.

We used the full adjacency matrix that contains the correlations between all pairs of ROIs to investigate if spatial smoothing has different effects on links of different weight and physical length. To this end, we defined the physical length of a link as the Euclidean distance between the centroids of the ROIs connected by the link.

For further analysis, we thresholded the adjacency matrix to remove weak links. Low‐weight links correspond to correlations that are too weak to be of functional significance; further, retaining only a small number of the strongest connections provides a view on the most essential network structure. To obtain a network with density *d*, we removed links that were weaker than the 1 − *d*th weight percentile by setting the corresponding element in the adjacency matrix to 0. The weights of links that exceeded this threshold were set to 1, which yielded an unweighted network.

We used the unweighted networks to study how spatial smoothing affects the degrees and eigenvector centralities of nodes, as these measures have originally been defined for unweighted networks. Both measures are commonly used to identify the most important, central nodes of the network (see below). Further, we analyzed the effects of spatial smoothing on the structure of the LCC (see below) of the unweighted network.

It is not straightforward to choose the optimal threshold density for functional brain network analysis (for discussion, see Kujala *et al*., 2016). One alternative is to investigate a range of densities (Alexander‐Bloch *et al*., 2012a; Lord *et al*., 2012). We found that the behavior of the centrality measures was qualitatively the same across a range of densities from 5 to 10%; the results reported in this article are obtained at the density of 10% (456 links). For analysis of the LCC, we thresholded the network to a lower density of 3% (137 links); at this density, we observed that there is a component that is clearly larger than all others without yet spanning the entire network. At both densities, the network was relatively sparse and therefore its structure was sensitive to small changes in link weights.

### Averaging and comparison of correlation coefficients

To investigate how spatial smoothing affected links of different weight and physical length, we needed to average correlation coefficients over subjects. This averaging of ROI–ROI correlations was done by first Fisher *Z*‐transforming the correlation coefficients *r*:

(3) $$Z=\frac{1}{2}\ln\left(\frac{1+r}{1-r}\right)=\text{arctanh}\;\left(r\right)$$

The results were then averaged, and finally the values were inversely transformed back to the interval [−1, 1]. The *Z*‐transformation reduces bias when averaging correlation coefficients (Silver & Dunlap, 1987).

To measure the difference between two correlation coefficients, they were first *Z*‐transformed and then subtracted.

### Centrality measures

We studied how spatial smoothing affects two measures of node centrality: the degree and the eigenvector centrality. Both measures are commonly used among neuroscientists to identify the most central and important nodes, *hubs*, of functional brain networks.

The degree of a node is defined as the number of neighbors of the node, that is, the number of other nodes it is directly connected with. The degree provides a simple estimate of the centrality of the node: nodes with many neighbors can be considered more central than those with few neighbors. Therefore, the degree can be used to define the hubs of the functional brain network (Rubinov & Sporns, 2010).

However, changes in the degree alone do not tell how spatial smoothing has changed the centrality of the node as compared to others. Therefore, to investigate if spatial smoothing changes the ‘hubness’ of nodes, we observed changes in both the degree and the degree rank of each ROI. To obtain the degree rank, we ordered ROIs in descending order by degree; changes in the degree rank of a ROI reflect changes in its ‘hubness’ in the network.

Eigenvector centrality is a generalization of degree that also takes into account the degrees of the node's neighbors, their neighbors, and so on. A high‐degree node can be argued to be less central if its neighbors have low degrees compared to another node with high‐degree neighbors. Eigenvector centrality corrects for this and measures how central the node is in this broader view. In functional brain networks, eigenvector centrality emphasizes the central clusters of the network (Lohmann *et al*., 2010).

Eigenvector centrality can be calculated iteratively from the adjacency matrix of the network. For details, see Appendix S1

For investigating the centrality metrics, we wrote an in‐house Python script that utilizes the NetworkX network analysis package for Python (Hagberg *et al*., 2008).

### Largest connected component

For a broader picture of changes in network structure beyond the level of nodes, we investigated how spatial smoothing affects the LCC of the network. In a (connected) component, every node can be reached from every other node by following links. In a sparse network, there can be several disjoint components; if the existence of a link is taken as indicative of functional interaction, nodes in disjoint components cannot influence one another. The LCC, the component with the largest number of nodes, can be seen as the core of the network.

First, we identified the LCC separately for the network of each subject. Then, for each node, we calculated the fraction of subjects that had that particular node in their LCC.

For studying changes in the LCC, we used a set of in‐house Python scripts (see above).

### Network visualization

The HO atlas offers anatomical coordinates of the centroids of the ROIs. We used the projection of these coordinates to the horizontal plane to visualize the network structure and the values of network metrics in each ROI. We made minor adjustments to the coordinates to avoid overlap between ROIs while approximately retaining the anatomical position of each ROI.

### ABIDE data

To ensure that our results are not explained by any particular feature of our in‐house dataset and can be generalized to other datasets, we repeated all analysis using a second, independent dataset. This dataset, to which we will now on refer as the ABIDE data, is part of the Autism Brain Imaging Data Exchange I (ABIDE I) project (Di Martino *et al*., 2014). It contains resting‐state fMRI of 28 healthy controls. Of these subjects, 19 were measured at California Institute of Technology (Caltech; 4 female, age 28.9 ± 11.2 years, mean ± SD) and 9 subjects at Carnegie Mellon University (CMU; all male, age 27.1 ± 6.5 years, mean ± SD). Subjects from the both measurement sites were pooled to form a single dataset.

The subjects of the ABIDE dataset were selected based on two criteria. First, we wanted to exclude children younger than 17 years since our in‐house dataset does not contain children and the brain networks are known to change by age. Second, to pool the subjects together, their data needed to be acquired with the same repetition time (TR), which in this case was 2.0 s. We picked across ABIDE I measurement sites the maximum number of adult subjects with a TR relatively close to the one used for collecting the in‐house dataset (1.7 s).

For further details of the ABIDE data, including the subject IDs and detailed data acquisition parameters, see Appendix S1

## Results

### Spatial smoothing increases weights of short links

**Figure 1:**
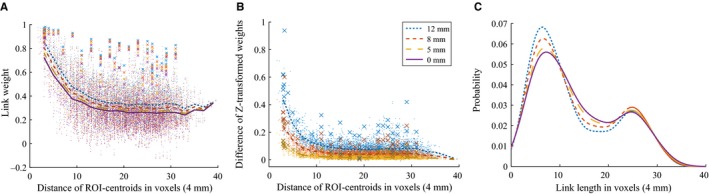
Spatial smoothing makes short and strong links stronger. (A) Regions of Interest (ROIs) that are close to each other have, on average, higher correlations, as seen from the dependence of link weights on the distance of the centroids of the ROIs. Smoothing increases the majority of the correlations. ROI–ROI correlations are averages over 13 subjects. Lines are bin averages and crosses mark correlations between the same areas in the different hemispheres. Colors and line types indicate smoothing kernel size (see panel B for legend). (B) Link weights at each smoothing level are compared to weights obtained from non‐smoothed data as a function of distance. Intuitively, the weight of short links increases more than the weight of long‐distance links. For calculating the differences in link weight, Fisher's *Z*‐transform has been used (see Methods section for details). (C) Distributions of link lengths in network thresholded at 10%. [Colour figure can be viewed at wileyonlinelibrary.com].

As functional brain networks are commonly built using Pearson correlation coefficients of ROI time series as link weights between ROIs and then thresholded, their structure is sensitive to any preprocessing method that affects link weights non‐uniformly. To see if spatial smoothing has similar effects on all links, we investigated the weight of each link as a function of its physical length. The strongest links of the network are physically short (Fig. 1A), as expected on the basis of earlier studies (Alexander‐Bloch *et al*., 2012b; Stanley *et al*., 2013). The only exception to this observation is the strong links connecting the same ROIs in opposite hemispheres (say, left and right frontal poles) that have also been reported in the literature (Anderson *et al*., 2010). Short links are affected by spatial smoothing more strongly than longer links: the weights of short links increase the most (Fig. 1B). Therefore, spatial smoothing alters the distributions of link lengths in thresholded networks (Fig. 1C; *d *=* *10%) and may change which links are included.

The reason for the weight increase of short links is that spatial smoothing mixes signals across ROI boundaries, between close voxels that belong to neighboring ROIs. This mixing is limited in range to the kernel width, and therefore voxels that are separated by longer distances are not directly affected.

### Spatial smoothing increases the degrees of small ROIs

**Figure 2:**
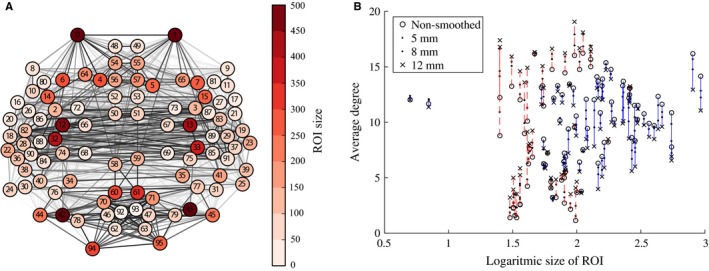
Spatial smoothing has different effects on Regions of Interest (ROIs) of different size. (A) Locations and sizes of the 96 ROIs in the HarvardOxford parcellation. Locations are slightly shifted from anatomical locations to avoid overlap. Many small ROIs are located in temporal lobes or surrounding areas. (B) Spatial smoothing increases the degrees of small ROIs (red dashed lines) whereas the degrees of larger ROIs decrease (blue solid lines) with the increasing full width at half maximum of the smoothing kernel. ROI degrees are averaged across 13 subjects. [Colour figure can be viewed at wileyonlinelibrary.com].

Next, we investigated whether the effects of smoothing on nodes are uniform. As the ROI sizes vary widely in the HO parcellation used here (Fig. 2A), we ask if spatial smoothing affects ROIs of different size differently.

As stated above, smoothing increases the weights of links between spatially adjacent ROIs. The signal of each ROI is a mixture of the signals of voxels in the interior of the ROI and the mixed voxel signals originating from the ROI boundary area (i.e., voxels adjacent to at least one voxel in a different ROI). In small ROIs, this boundary area is relatively large when compared to larger ROIs. This suggests that spatial smoothing has different effects on the network connectivity of small and large ROIs.

We test this hypothesis by investigating the degrees of ROIs, that is, their numbers of network neighbors, in networks thresholded to 10% density. Because of thresholding, the degree is determined by link weights: only links with high enough weight pass the threshold and contribute to node degree. Figure 2B displays the degrees as a function of ROI size when different levels of spatial smoothing are applied. As expected, the degrees of small ROIs are increased: signal mixing across ROI boundaries leads to higher weights of links of small ROIs. As the total number of links in the thresholded network is constant, the degrees of larger ROIs decrease as the width of the smoothing kernel increases. The Pearson correlation coefficient between ROI size and degree change quantifies this observation (FWHM5: *r *= −0.55, *p* ≪ 10^−5^; FWHM8: *r *= −0.53, *p *< 10^−5^; FWHM12: *r *= −0.57, *p *< 10^−5^): degrees of small ROIs increase, while for large ROIs the degree change is negative.

### Network centrality measures are distorted by spatial smoothing

**Figure 3:**
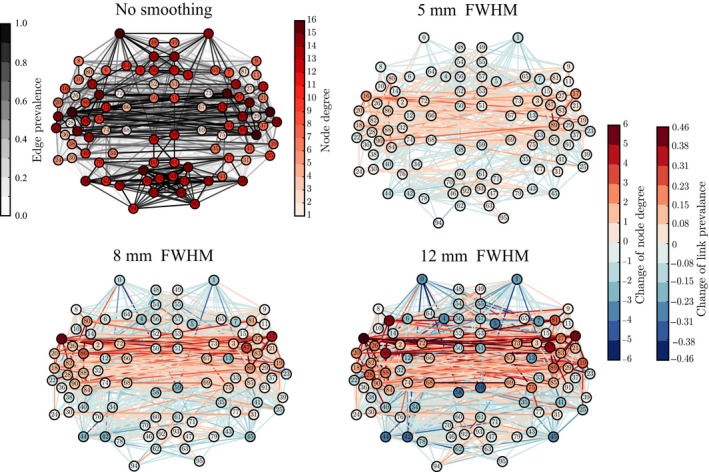
The changes in degree due to spatial smoothing are concentrated around temporal lobes. The top left panel shows the degrees of the Regions of Interest in the network obtained without smoothing. The other panels display differences between the network for non‐smoothed data and networks resulting from smoothing the voxel‐level signals using kernels with different full width at half maximum (FWHM) (5, 8, and 12 mm). The degree values of the non‐smoothed network are subtracted from the degree values of the smoothed networks; red (blue) color indicates increase (decrease) of degree in the smoothed network. The colors of links indicate the change in prevalence, that is, fraction of subjects, of 13, that had a given link present in their thresholded network. Networks are thresholded to 10% link density. All degrees are averages over the networks of 13 subjects. [Colour figure can be viewed at wileyonlinelibrary.com].

**Figure 4:**
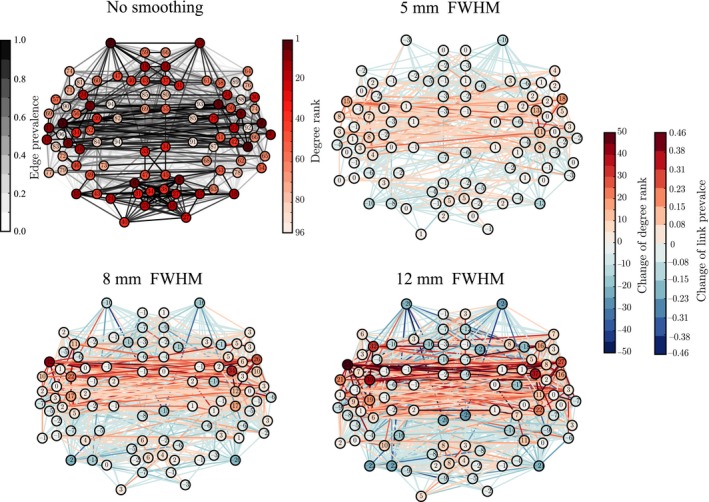
The ‘hubness’ of the nodes in the temporal lobes, measured in terms of their degree ranks, is increased by smoothing. The values of ranks and rank changes are shown as node labels. The networks corresponding to smoothing kernels of full width at half maximum (FWHM) 5, 8, and 12 mm display differences as compared to the network for non‐smoothed data, similarly to Fig. 3. [Colour figure can be viewed at wileyonlinelibrary.com].

**Figure 5:**
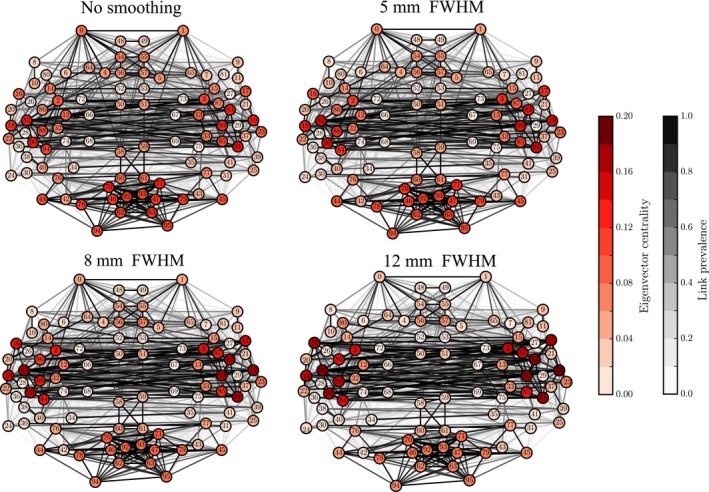
Changes in eigenvector centralities of nodes due to smoothing reflect the changes in degree and degree ranks, as the largest increase is concentrated around the temporal lobes. In contrast to Figs 3 and 4, node and link colors indicate absolute values of eigenvector centrality and link prevalence instead of differences. [Colour figure can be viewed at wileyonlinelibrary.com].

Many network properties of nodes are heavily affected by node degree. Because smoothing has non‐uniform effects on the degrees of ROIs, we expect to see non‐uniform changes in other network properties as well. We focus on the relationship between network properties and the anatomical layout of the ROI network. For each ROI, we computed the most commonly used centrality measures: degree as above (Fig. 3), degree rank (Fig. 4), and eigenvector centrality (Fig. 5), for different levels of smoothing.

In a network thresholded to a fixed density (here, 10%), spatial smoothing cannot change the mean degree. However, it affects the shape of the degree distribution: while both the maximum and minimum degrees increase with smoothing, the median degree decreases, which increases the skewness of the distribution (for details, see Appendix S2 and Table S1).

This indicates that there are more ROIs with decreasing than with increasing degree, that is, the degree of a few ROIs increases and the degrees of most of other ROIs decreases to compensate for this change. Indeed, when we visualized the values of these measures at locations corresponding to the anatomical coordinates of the ROI centroids (Fig. 3), the largest changes were observed in some ROIs in the temporal lobes and their vicinity. In contrast, connections were weakened or lost between most other areas.

The degree and eigenvector centrality were strongly correlated at all levels of smoothing (Pearson correlation coefficient FWHM0: *r *=* *0.91, *p* ≪ 10^−5;^ FWHM5: *r *=* *0.91, *p* ≪ 10^−5^; FWHM8: *r *=* *0.90, *p* ≪ 10^−5^; FWHM12: *r *=* *0.90, *p* ≪ 10^−5^). Therefore, it is not surprising that we obtained mostly similar results as for degree (Fig. 5; for details, see Appendix S2 and Table S1).

Spatial smoothing dramatically changes the ‘hubness’ of some nodes (Fig. 4): the degree ranks of some temporal, frontal, and parietal ROIs increased while they decreased for some occipital ROIs and midline regions as well as for the frontal poles. In particular, the degree ranks increased for the left and right superior temporal gyrus (anterior division), left and right middle temporal gyrus, left frontal operculum gyrus, right parietal operculum gyrus, and left and right planum polare. Areas that decreased most in degree rank included the left and right frontal pole, right superior frontal gyrus, superior and inferior division of left lateral occipital cortex, inferior division of right lateral occipital cortex, and anterior division of left and right cingulate gyrus.

Regions of interest that are hubs of the network are often assumed to be central in the transfer and processing of information during the given task. Because of this, the observed changes in the degree ranks may dramatically change the interpretation of the functional roles of different ROIs in the network.

### Smoothing may disconnect brain areas in the functional networks

**Figure 6:**
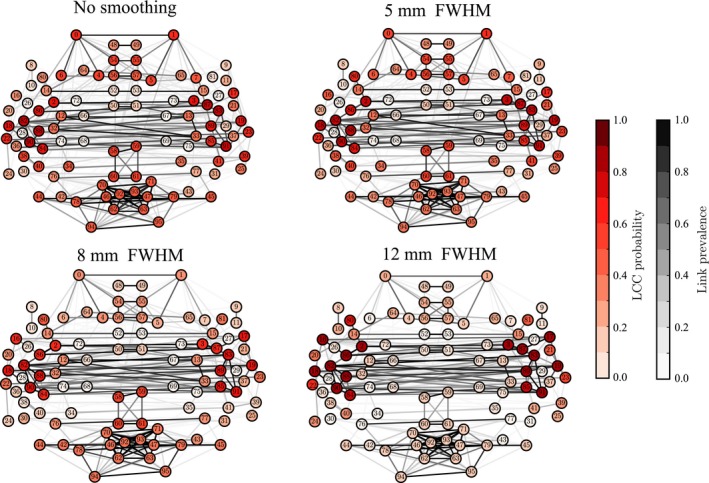
Spatial smoothing disconnects the frontal and occipital Regions of Interest (ROIs) from the largest connected component (LCC). Node colors indicate how frequently a certain ROI belongs to the LCC in the networks of 13 subjects. Networks are thresholded to 3% density. [Colour figure can be viewed at wileyonlinelibrary.com].

So far, we have investigated the effects of spatial smoothing on single nodes and links. Next, we ask whether spatial smoothing also changes the overall structure of the functional network. To this purpose, we extracted the LCCs of the networks at low density (3%, 137 links) (Fig. 6; see Methods). The LCC forms the functional core of the network; purely from the network point of view, all flows of information are constrained to take place within connected components only. Therefore, if some set of nodes drops out from the LCC, the interpretation of its functional role changes dramatically.

In particular, we investigated the probability of nodes to belong to the LCC across subjects, that is, the fraction of subjects that have the ROI in their LCC. Without spatial smoothing, a small number of ROIs had a high probability of belonging to the LCC. At the same time, many nodes had moderate probabilities only and were spatially spread out, indicating a high level of variation in network structure across subjects. However, spatial smoothing decreased this variation: for a FWHM of 12 mm, the LCCs of virtually all participants comprised ROIs from the temporal lobes and neighboring areas. To the contrary, the occipital and frontal ROIs that in the absence of smoothing had nonzero LCC probabilities were cut out from the LCC when smoothing was applied.

The significance of this result is that when the network is thresholded to low density, retaining only the strongest connections, smoothing can change which parts of brain networks are included in the largest component. With higher densities, almost all nodes belong to the LCC, and smoothing only affects how strongly different areas are connected.

## Discussion

### Spatial smoothing changes the identity of hubs of the functional brain network

In this study, we have investigated whether the use of spatial smoothing alters the structure of functional brain networks. It has been known that smoothing affects the properties of voxel‐level networks, but these effects have been suggested to be smaller at the ROI level (van den Heuvel *et al*., 2008; Zalesky *et al*., 2012). However, our results with two independent data sets show that spatial smoothing has significant effects on ROI‐level networks built using the anatomical HO atlas, resulting in artefacts and network features that depend on the parameters of the applied smoothing process. While increased numbers of subjects or additional data sets might reveal detailed effects of smoothing on particular nodes or links, our results clearly point out that spatial smoothing has unwanted side effects on functional brain networks.

Applying spatial smoothing means that voxel signals are averaged, both within and across the boundaries of ROIs. Therefore, smoothing makes the time series of ROIs more similar. However, the effects of smoothing are not limited to increasing the correlation between time series of physically adjacent ROIs. Rather, smoothing has also highly nontrivial effects at the level of the whole network.

In particular, we have observed increased degree and eigenvector centrality in small ROIs. Because the degree and eigenvector centrality are commonly used to identify the hubs of functional brain networks (Rubinov & Sporns, 2010; Zuo *et al*., 2012), this means that different nodes may be seen as hubs depending on whether smoothing is applied or not.

As smoothing increases the centralities of small ROIs, they are more likely to be considered as hubs. This is at least partially explained by their size: small ROIs have large boundary area relative to their volume, and therefore their time series are the ones that are most affected when voxel signals originating at different ROIs are mixed by smoothing.

### Spatial smoothing changes the structure of functional brain networks

Because spatial smoothing increases the correlation between the time series of ROIs, link weights are generally increased in the ROI network. However, this effect is not uniform across the links: the weights of physically short links increase the most, while longer links are less affected. Therefore, when the network is thresholded to a constant density, smoothing increases the proportion of short links.

Short links are typically located in tightly connected clusters of brain areas. To adopt the terminology of Tononi *et al*. (1994) and Sporns (2013b), spatial smoothing increases the segregation and decreases the integration of functional brain networks: connectivity within local clusters is increased whereas connections between such clusters get weaker. Yet effective information processing in the brain requires both integration and segregation (Sporns, 2013b), and especially the longer and weaker links between local clusters are of crucial importance (Gallos *et al*., 2012). It is exactly these links whose strength and importance are downplayed by spatial smoothing.

### Spatial smoothing decreases inter‐subject variation

The LCC presents the core of the functional brain network. In non‐smoothed data, LCCs differ between the networks of different subjects, whereas after smoothing the LCCs are more similar for all subjects. In other words, spatial smoothing reduces the diversity between subjects. This may be a desired effect in some studies. However, is there really a core of the functional brain network that is universal across subjects? After spatial smoothing, ROIs may belong to the LCC not because of their true centrality but because of the side effects of smoothing.

The decrease of inter‐subject variation raises the question whether variation is also decreased between subject groups that are supposed to be different, for example, patients and controls. This should be tested in further experiments, where the differences in network structure between two groups are measured with and without spatial smoothing. Unfortunately, a comprehensive comparison is made difficult by the lack of a general way of measuring differences between networks; typically, they are quantified with methods that have been tailored for each study.

### Is spatial smoothing necessary for ROI‐level functional networks?

Spatial smoothing is often used as a part of the preprocessing pipeline prior to functional brain network analysis partly for historical reasons: the advantages of spatial smoothing in standard GLM analysis are beyond dispute and smoothing therefore belongs to the standard set of fMRI preprocessing tools (Mikl *et al*., 2008). Further, the effects of spatial smoothing, mainly the increase of SNR due to averaging voxel signals, have been thought to be advantageous in network neuroscience as well.

It is worth noting that in the ROI approach, a lot of signals are averaged whether smoothing is applied or not, to form the time series that represent ROI activity. Further, unlike spatial smoothing, this averaging does not mix signals that belong to different ROIs. Therefore, one may question what advantage could be gained by the additional averaging due to spatial smoothing.

## Conclusion

ROI‐level resting‐state functional brain networks are affected by spatial smoothing. Spatial smoothing has complex effects on the structure and properties of the networks, including possible over‐emphasis of strong, short‐range links, changes in the identities of hubs of the network, and decreased inter‐subject variation. The ROI approach already includes averaging, independent of spatial smoothing. Therefore, there is no specific reason for applying spatial smoothing.

## Competing interests

Authors reported no conflicts of interest.

## Author contributions

TA, OK, and JS designed the study. HS supplied data. TA, EG, and OK contributed tools for preprocessing and analysis. TA performed the analysis. TA, OK, and JS wrote the manuscript with the help of comments from all authors.

## Data accessibility

The ethical restrictions of our Ethical Committee permission and the Finnish law prohibit sharing of biomedical data measured on human individuals, anonymized or not. Therefore, we are not allowed to make the in‐house dataset used in this study publicly available. The data are stored in Brain and Mind Laboratory, Aalto University, Espoo, Finland, and the authors will, by request, provide assistance for reproducing the results reported in this article on the original dataset.

**Abbreviations**
ABIDE: Autism Brain Imaging Data Exchange
BOLD: blood‐oxygen‐level dependent
CSF: cerebrospinal fluid
EPI: echo planar imaging
fMRI: functional magnetic resonance imaging
FOV: field of view
FWHM: full width at half maximum
GLM: general linear model
HO: HarvardOxford
LCC: largest connected component
MR: magnetic resonance
ROI: Region of Interest
SNR: signal‐to‐noise ratio
TE: echo time
TR: repetition time

## Supporting information

Appendix S1. Supplementary Methods: algorithm for calculating eigenvector centrality; details of the ABIDE data.
Appendix S2. Supplementary Results: Analysis repeated for the ABIDE data. Figures below visualize these results.
Fig. S1. Short, strong links get stronger when spatial smoothing is applied.
Fig. S2. Spatial smoothing increases degrees of small ROIs.
Fig. S3. Spatial smoothing increases degrees of temporal and occipital ROIs.
Fig. S4. Spatial smoothing increases the “hubness”, measured by degree rank, of ROIs in temporal and occipital lobes.
Fig. S5. Spatial smoothing changes eigenvector centrality values of nodes.
Fig. S6. Spatial smoothing changes the structure of the LCC.
Table S1. Names and sizes of ROIs and detailed numerical results about the effects of spatial smoothing.
