# Supplementary material for: Effects of spatial smoothing on functional brain networks
Source: Eur J Neurosci. 2017 Oct 20;46(9):2471–80. doi: 10.1111/ejn.13717 (PMC5698731; doi:10.1111/ejn.13717)
Supplement: Supplementary file 2 — Appendix S1. Supplementary Methods: algorithm for calculating eigenvector centrality; details of the ABIDE data. Appendix S2. Supplementary Results: Analysis repeated for the ABIDE data. Figures below visualize these results. Fig. S1. Short, strong links get stronger when spatial smoothing is applied. Fig. S2. Spatial smoothing increases degrees of small ROIs. Fig. S3. Spatial smoothing increases degrees of temporal and occipital ROIs. Fig. S4. Spatial smoothing increases the “hubness”, measured by degree rank, of ROIs in temporal and occipital lobes. Fig. S5. Spatial smoothing changes eigenvector centrality values of nodes. Fig. S6. Spatial smoothing changes the structure of the LCC. Table S1. Names and sizes of ROIs and detailed numerical results about the effects of spatial smoothing. [file EJN-46-2471-s002.pdf]

Supplementary Information for Effects of spatial smoothing on functional brain networks

Tuomas Alakörkkö, Heini Saarimäki, Enrico Glerean, Jari Saramäki, Onerva Korhonen

1 Supplementary Methods

1.1 Iterative algorithm for calculating eigenvector centrality

We calculate the eigenvector centrality with an iterative algorithm utilizing the adjacency matrix of the network. First, eigenvector centrality of each node of the network is initialized to 1. Then, at each timepoint t the eigenvector centrality of the focal node i is defined as

$$c_i^{\mathrm{eig}}(t) = \sum_{j=1}^{N} a_{ij} c_j^{\mathrm{eig}}(t-1), \tag{1}$$

where N is the total number of nodes in the network, a_{ij} is the element of the adjacency matrix $\boldsymbol{A}$ that corresponds to the link between nodes i and j, and $c_j^{\mathrm{eig}}(t-1)$ denotes the eigenvector centrality of node j at timepoint $t-1$. We can write this in the matrix form:

$$\boldsymbol{c}^{\mathbf{eig}}(t) = \boldsymbol{A}\boldsymbol{c}^{\mathbf{eig}}(t-1) = \boldsymbol{A}^t \boldsymbol{c}^{\mathbf{eig}}(0). \tag{2}$$

Next, we can express this in terms of eigenvectors $\boldsymbol{v_i}$ and eigenvalues λ_i of $\boldsymbol{A}$:

$$\boldsymbol{c}^{\mathbf{eig}}(t) = \lambda_1^t \sum_{i=1}^{N} b_i \left[\frac{\lambda_i}{\lambda_1}\right]^t \boldsymbol{v_i}, \tag{3}$$

where λ_1 is the largest eigenvalue of $\boldsymbol{A}$ and b_is are a set of multipliers. Now, when time increases to infinity,

$$\lim_{t\to\infty} \boldsymbol{c}^{\mathbf{eig}}(t) = \lambda_1^t b_1 \boldsymbol{v_1}. \tag{4}$$

So, after normalization, the eigenvector centrality vector is the eigenvector that corresponds to the largest eigenvalue of the adjacency matrix.

1.2 ABIDE data

In order to ensure that the results obtained in the present article are not caused by any feature unique to our in-house dataset, we repeated all the analysis for a second, independent dataset. This dataset has been published as a part of the Autism Brain Imaging Data Exchange I (ABIDE I) initiative (Di Martino et al., 2014). From now on, we will refer to this dataset as the ABIDE data.

Subjects

The abide dataset contains the data of 28 healthy control subjects. These subjects were measured as a part of the ABIDE I initiative; 19 subjects at California Institute of Technology (Caltech) and 9 subjects at Carnegie Mellon University (CMU). In the present study, subjects from both measurement sites were pooled in order to form a single dataset.

The subjects of the ABIDE dataset were selected based on two criteria. First, we wanted to exclude from the dataset all children younger than 17 years. The networks of the human brain are known to change by age, and since our in-house dataset does not contain any children, including them into the ABIDE dataset may had caused uncontrollable differences between the datasets. Second, in order to pool the subjects together, their data needed to be collected with a single repetition time (TR), preferably with one relative close to the one used for collecting the in-house dataset (1.7s). So, the selected subjects are the largest possible subset of the adult ABIDE I subjects collected with a TR of 2.0s.

ABIDE subject IDs of the 19 subjects measured at Caltech were 51475, 51476, 51477, 51478, 51479, 51480, 51481, 51482, 51483, 51484, 51485, 51486, 51487, 51488, 51489, 51490, 51491, 51492, and 51493. Out of these subjects, 15 were male and 4 female. Their age ranged between 17 and 56.2 years with the mean of 28.9±11.2 years (mean±SD). 15 of the subjects were right-handed and one left-handed, while the handedness of 3 subjects was ambiguous. The subjects reported no history of either autism spectrum disorders (ASD) or other psychiatric or neurological disease nor family history of ASD. The Caltech data has been described in detail in Tyszka et al. (2014).

ABIDE subject IDs of the 9 subjects measured at CMU were 50657, 50659, 50660, 50663, 50664, 50665, 60666, 60667, and 50668. All the CMU subjects were male. Their age varied between 21 and 40 years with the mean of 27.1±6.5 years (mean±SD). 8 of them were right-handed and one had ambiguous handedness. None of these subjects had reported history of ASD or other psychiatric or neurological disease.

Data acquisition

The MRI and fMRI data of the subjects measured at Caltech were acquired with a 3 Tesla Magnetom Trio device (Siemens Medical Solutions, NJ, USA). Structural MR images were acquired with a T1-weighted MP-RAGE sequence with $1 \times 1 \times 1\text{mm}^3$ isotropic voxel size. The resting-state fMRI data were measured as a whole-head T2*-weighted EPI sequence with the following parameters: TR = 2.0 s, TE = 30 ms, flip angle = 75 °, voxel size = $3.5\times3.5\times3.5\text{mm}^3$,

matrix size = 64 x 64 x 34, FOV = 256 x 256 mm^2. The length of the time series was 3 minutes (150 time points). During the measurement, the subjects were instructed to lie still and keep their eyes closed but prevent falling asleep.

For subjects measured at CMU, MRI and fMRI data were acquired with a 3 Tesla Magnetom Verio device (Siemens Medical Solutions, NJ, USA). Structural MR images were acquired with a T1-weighted MP-RAGE sequence with isotropic voxel size of $1 \times 1 \times 1\text{mm}^3$. The resting-state fMRI data were measured with a whole-head T2*-weighted EPI sequence with the following parameters: TR = 2.0 s, TE = 30 ms, flip angle = 73 °, voxel size = $3.0 \times 3.0 \times 3.0\text{mm}^3$, matrix size = 64 x 64 x 20, FOV = 192 x 192 mm^2. The length of the time series was 10.4 min (320 time points). During the scanning, subjects lay still with their eyes closed in a room with lights shut off.

Preprocessing and analysis

The preprocessing and analysis pipeline of the ABIDE dataset was identical to the one applied on our in-house dataset. For details, see the Methods section of the main article. The measurement parameters slightly differed between the Caltech and CMU subjects. Therefore, in order to avoid any unpredictable effects caused by differences in data acquisition, the anatomical MR images of all subjects were registered to MNI152 standard template and resampled to voxel size of $4 \times 4 \times 4\text{mm}^3$ prior to creating the group-level masks for obtaining ROIs. Further, time series of different subjects – and possibly of different lengths – were never averaged during the analysis. Instead, functional brain networks were extracted separately for each subject, and all averaging over subjects was done only after extracting the networks.

Note that the number of ROIs differend between our in-house dataset and the ABIDE data: the ABIDE data contained only 92 anatomical ROIs. This is because the locations of ROIs 27, 28, 73, and 74 (left and right inferior temporal gyrus, anterior division, and left and right temporal fusiform cortex, anterior division) did not overlap across all ABIDE subjects. In the ABIDE data, the ROI size varied between 5 and 765 with mean size 127.07±124.18 (mean±std). The median ROI size was 86.5.

2 Supplementary Results

2.1 Changes in centrality distributions

In the main article (section), we noticed that spatial smoothing alters the shape of the degree distribution in our in-house dataset. In a network thresholded to a fixed density, spatial smoothing cannot change the mean degree. Instead, the median and standard deviation of degree decreased with the increasing level of smoothing (FWHM0: $k = 10.19$, std = 4.22; FWHM5: $k = 10.04$, std = 4.19; FWHM8: $k = 9.62$, std = 4.17; FWHM12: $k = 9.19$, std = 4.12). Meanwhile, both the minimum and maximum degrees increased (FWHM0: $k_{min} = 1.15$, $k_{max} = 16.15$; FWHM5: $k_{min} = 1.54$, $k_{max} = 16.92$; FWHM8: $k_{min} = 2.31$, $k_{max} = 17.92$; FWHM12: $k_{min} = 3.23$, $k_{max} = 19.08$), and the number of degree values smaller than the mean increased. In summary, spatial smoothing increased the skewness of the degree

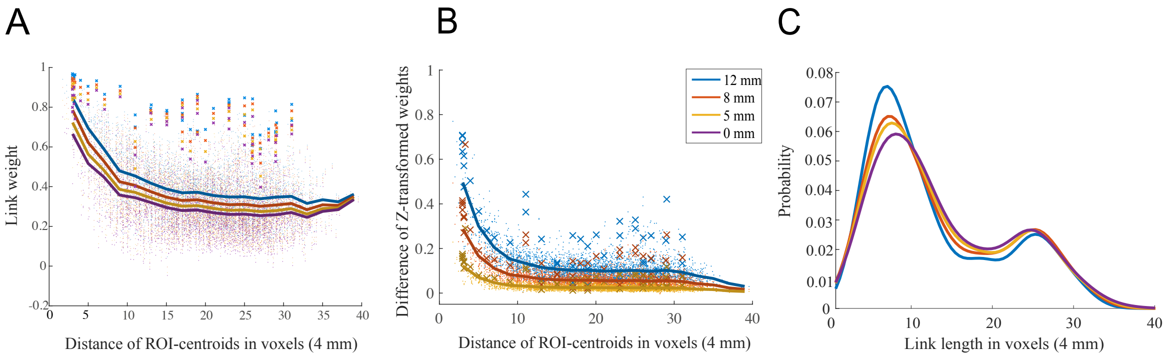

Figure 1: Short, strong links get stronger when spatial smoothing is applied. A) The strongest links connect ROIs that are physically close to each other. This dependency of the link weight and the physical length of a link is independent of spatial smoothing, although smoothing increases correlations between all ROIs. ROI-ROI correlations are averages over 28 participants. Lines show bin averages, and crosses mark correlations between the corresponding anatomical areas in the different hemispheres. Colors indicate smoothing kernel size (see panel B for legend). B) The increase in weight due to spatial smoothing is largest for short links. The change of link weight between each smoothing level and the non-smoothed case is calculated using Fisher's Z-transform (for details, see section in the main article). C) Spatial smoothing alters the distribution of the physical link length. Distributions have been calculated from the pooled data of 28 subjects in networks thresholded to $d = 10\%$.

distribution.

As one may expect, degree and eigenvector centrality were strongly correlated in our in-house dataset, and also changes in the distributions of degree and eigenvector centrality due smoothing were mostly the same. The median eigenvector centrality decreased with the increasing level of smoothing (FWHM0: $c^{\text{eig}} = 0.059$, FWHM5: $c^{\text{eig}} = 0.054$, FWHM8: $c^{\text{eig}} = 0.048$, FWHM12: $c^{\text{eig}} = 0.038$), and both the maximum and minimum eigenvector centrality increased (FWHM0: $c^{\text{eig}}_{max} = 0.077$, $c^{\text{eig}}_{min} = 0.0030$; FWHM5: $c^{\text{eig}}_{max} = 0.15$, $c^{\text{eig}}_{min} = 0.0030$; FWHM12: $c^{\text{eig}}_{max} = 0.17$, $c^{\text{eig}}_{min} = 0.0040$; FWHM12: $c^{\text{eig}}_{max} = 0.19$, $c^{\text{eig}}_{min} = 0.0050$). However, there were also some differences. First, as the mean of eigenvector centrality is not fixed by the network density, it decreased with increasing level of smoothing (FWHM0: $c^{\text{eig}} = 0.061$, FWHM5: $c^{\text{eig}} = 0.061$, FWHM8: $c^{\text{eig}} = 0.058$, FWHM12: $c^{\text{eig}} = 0.056$). Further, contrary to degree, the standard deviation of eigenvector centrality increased with smoothing (FWHM0: std = 0.038, FWHM5: std = 0.041, FWHM8: std = 0.046, FWHM12: std = 0.052).

2.2 ABIDE data

We started our analysis of the ABIDE data by investigating how spatial smoothing affects the links of the functional brain network, where the nodes depict Regions of Interest (ROIs) and links are defined as Pearson correlation coefficients between the time series of ROIs. As in the case of the in-house dataset, also in the networks extracted using the ABIDE data the strongest links were physically short (Fig. 1A). Spatial smoothing increased the weight of these short links more than the strength of longer links (Fig. 1B). This lead to an altered distribution of link lengths in thresholded networks (Fig. 1C; $d = 10\%$).

We continued by investigating if spatial smoothing changes the degree of the nodes of the

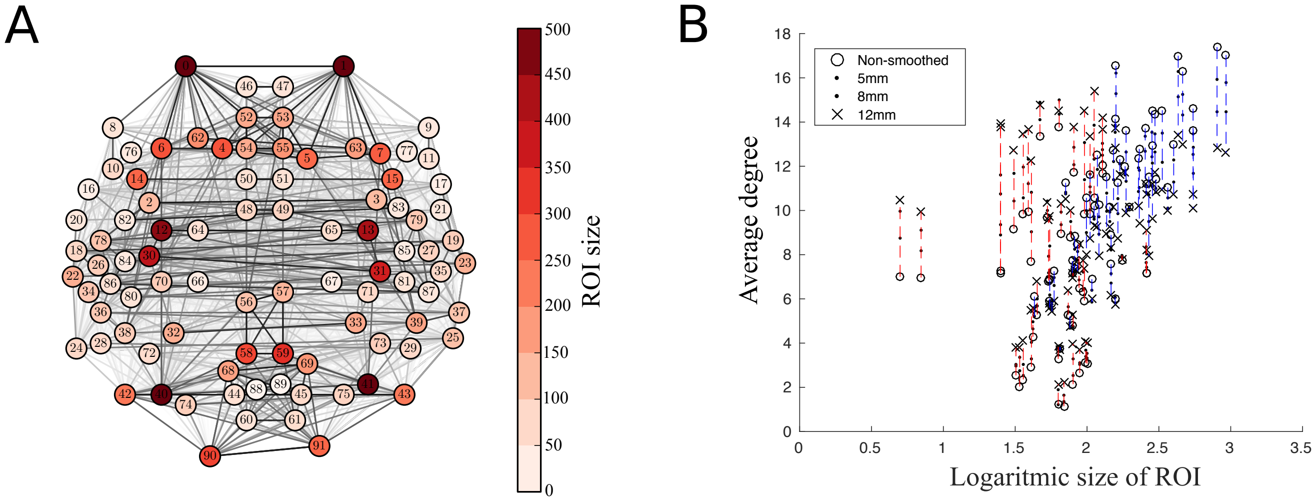

Figure 2: Spatial smoothing increases degrees of small ROIs. A) Locations and sizes of the 92 anatomical ROIs from the HO parcellation. Locations are slightly shifted from anatomical locations to avoid overlap. Many small ROIs are located in temporal lobes or surrounding areas. B) Spatial smoothing increases the degrees of small ROIs (red dashed lines) whereas the degrees of larger ROIs decrease (blue dashed lines). ROI degrees are averaged over 28 subjects.

functional brain networks. In the analysis of the in-house dataset, we have noticed that degrees of small ROIs, *i.e.* ROIs that contain few voxels, increased when smoothing is applied. This is true also for the ABIDE dataset: small ROIs gained new links in networks thresholded to $d = 10\%$ (Fig. 2). Meanwhile, the degree of larger ROIs decreased, since the number of links in the thresholded network must stay constant. The Pearson correlation coefficient between ROI size and degree change quantifies this observation (FWHM5: $r = -0.63$, $p < 10\ -5$; FWHM8: $r = -0.64$, $p < 10^{-5}$; FWHM12: $r = -0.65$, $p < 10^{-5}$): degree of small ROIs increased, while degree of larger ROIs decreased.

Node degree is commonly used to define the most central nodes, *hubs*, of the network. Therefore, the non-uniform changes in node degree due to spatial smoothing hint that smoothing possibly affects the hubs of the network. To investigate this in more detail, we calculated three commonly-used centrality measures: node degree (Fig. 3), degree rank (Fig. 4), and eigenvector centrality (Fig. 5) for each ROI. For detailed results, see Supplementary Table.

Similarly as in the in-house data, we obtained more negative than positive degree changes. The minimum degree increased and standard deviation decreased as a function of the width of the smoothing kernel (FWHM0: $k_{min} = 1.14$, FWHM5: $k_{min} = 1.21$, FWHM8: $k_{min} = 1.64$, FWHM12: $k_{min} = 2.14$). However, contrary to the in-house data, the median degree increased (FWHM0: $k = 9.07$, FWHM5: $k = 9.54$, FWHM8: $k = 9.73$, FWHM12: $k = 9.36$) and maximum degree decreased with the increasing level of smoothing (FWHM0: $k_{max} = 17.39$, FWHM5: $k_{max} = 16.28$, FWHM8: $k_{max} = 15.28$, FWHM12: $k_{max} = 15.39$. This indicates that the changes caused by spatial smoothing are nontrivial and may depend also on the properties of the dataset analyzed.

Degree and eigenvector centrality were strongly correlated also in the ABIDE dataset (Pearson correlation coefficient FWHM0: $r = 0.97$, $p \ll 10^{-5}$; FWHM5: $r = 0.96$, $p \ll 10^{-5}$; FWHM8: $r = 0.96$, $p \ll 10^{-5}$; FWHM12: $r = 0.94$, $p \ll 10^{-5}$). Mean and median of the eigenvector centrality decreased with the increasing level of smoothing similarly as in the in-house data (FWHM0: mean: $c^{\mathrm{eig}} = 0.066$, median: $c^{\mathrm{eig}} = 0.067$; FWHM5: mean: $c^{\mathrm{eig}} = 0.065$, median:

$c^{\text{eig}} = 0.068$; FWHM8: mean: $c^{\text{eig}} = 0.064$, median: $c^{\text{eig}} = 0.067$; FWHM12: mean: $c^{\text{eig}} = 0.061$, median: $c^{\text{eig}} = 0.059$). In standard deviation or minimum and maximum values of eigenvector centrality we found no clear, systematic changes (see Supplementary Table). Also the changes in the eigenvector centrality of single nodes were smaller than in the in-house data.

Similarly as in the in-house data, increase in degree rank was concentrated in temporal lobes and their vicinity. The areas, for which the degree rank increased most, included left and right superior temporal gyrus (anterior division), right superior temporal gyrus (posterior division), left and right central operculum cortex, left and right planum temporale, left and right Heschl's gyrus, and left supracalcarine cortex. Among the areas that decreased the most in degree rank were left and right middle frontal gyrus, left supramarginal gyrus (posterior division), right occipital gyrus (superior division), left lateral occipital cortex (inferior division), right cingulate gyrus (anterior division), and left and right cingulate gyrus (posterior division).

Finally, we asked if spatial smoothing changes the overall structure of the functional brain networks. In particular, we investigated the largest connected component (LCC) of the network. In the case of the in-house data, we noticed that spatial smoothing decreased the inter-subject variation in the structure of the LCC. Further, smoothing also significantly decreased the probability of some areas, especially occipital ROIs and frontal poles, to belong to the LCC. In the case of the ABIDE data, this effect is not as visible as in the in-house data (Fig. 6), although some decrease in probability of belonging to the LCC can be observed especially in the occipital lobe. One possible explanation for the differences in the effect size between the in-house data and the ABIDE data is the number of subjects (13 vs 28). Since the ABIDE dataset contains more subjects, the original inter-subject variance may be larger than in the in-house dataset and therefore better survive also after spatial smoothing.

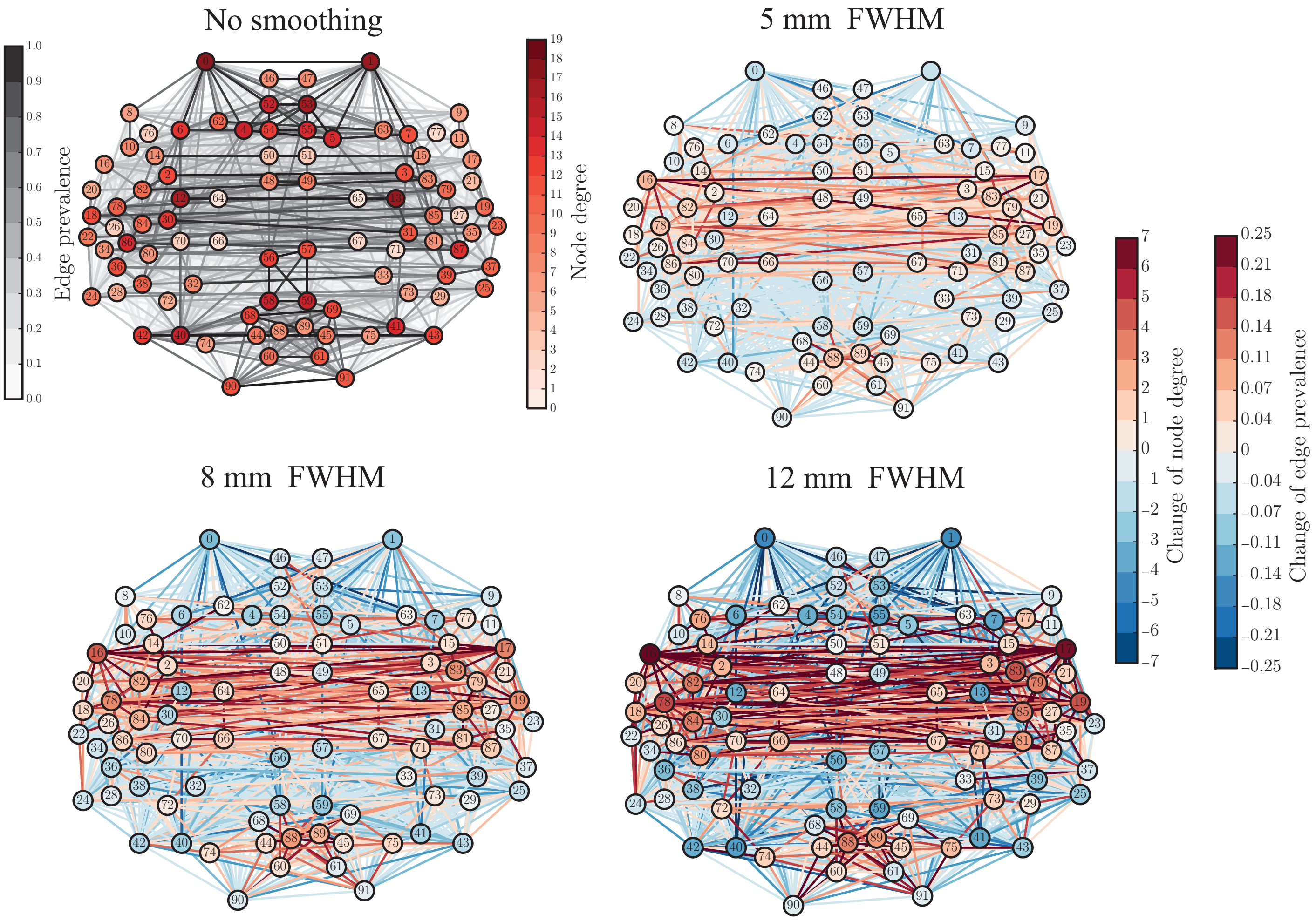

Figure 3: Spatial smoothing increases degrees of temporal and occipital ROIs. The top left panel shows, as a reference, the degrees of the ROIs in the network extracted from non-smoothed data. Rest of the panels display degree differences between the reference network and networks extracted from data that have been smoothed with different-sized kernels (FWHM of 5 mm, 8 mm, and 12 mm). Red (blue) colors indicate increase (decrease) of degree in networks extracted from smoothed data. The colors of links indicate the change in prevalence, *i.e.* fraction of subjects, out of 28, that had a given link present in their thresholded network. Networks are thresholded to 10% link density. All degrees are averages over the networks of 28 subjects.

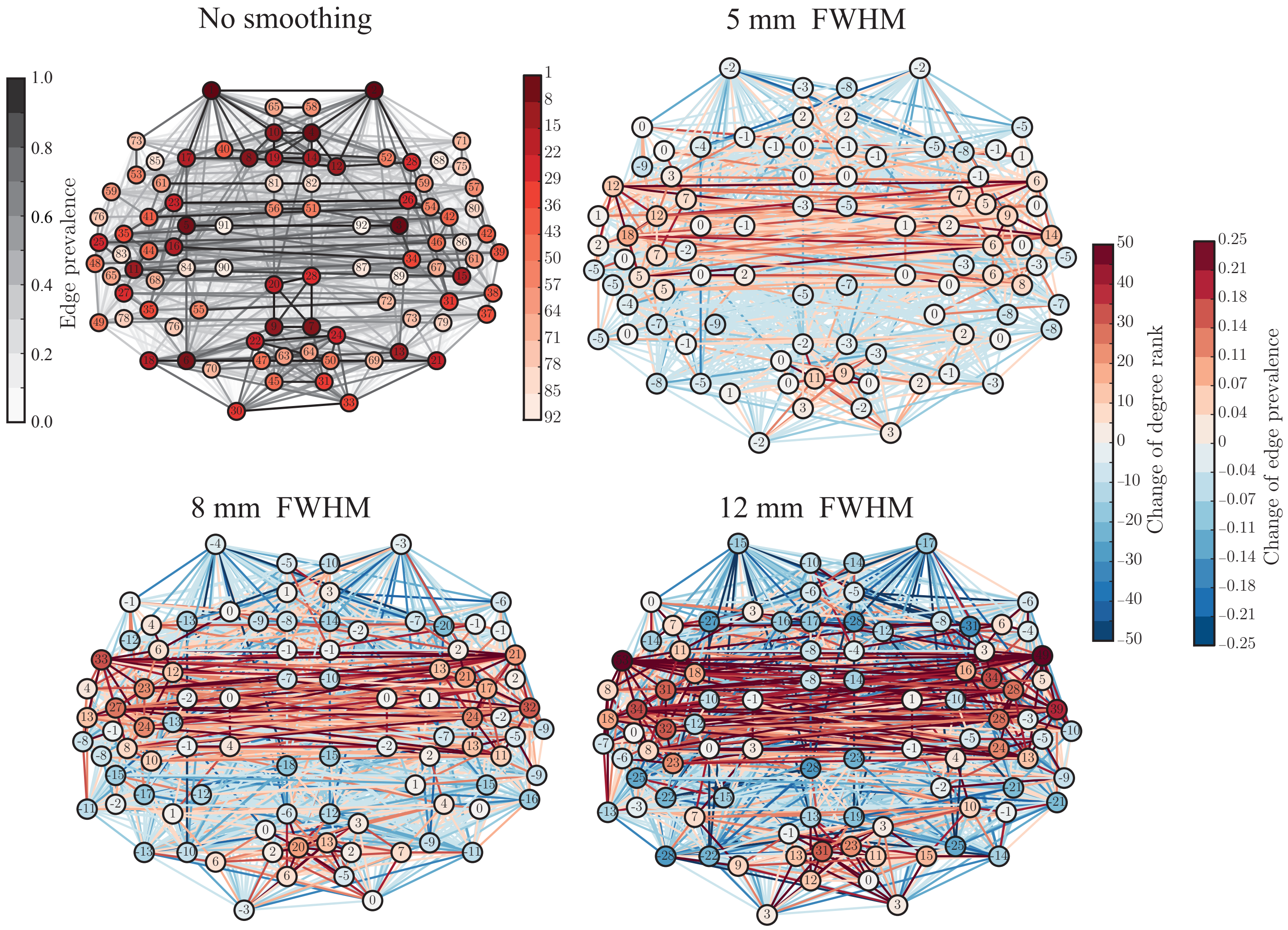

Figure 4: Spatial smoothing increases the "hubness", measured by degree rank, of ROIs in temporal and occipital lobes. The values of ranks and rank changes are shown as node labels. The networks corresponding to smoothing kernels of FWHM 5mm, 8mm, and 12mm display differences as compared to the network for non-smoothed data, similarly to Fig. 3.

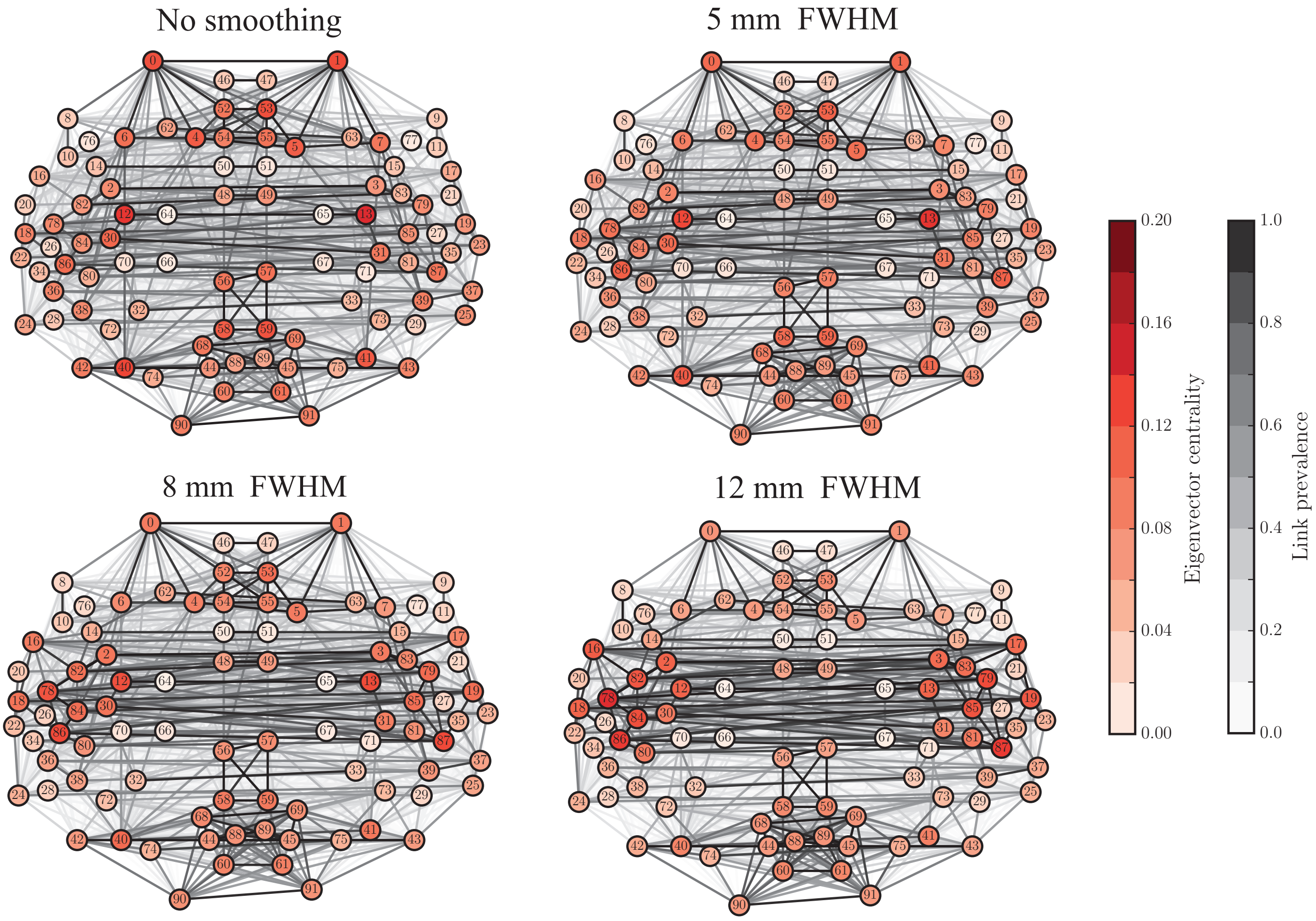

Figure 5: Spatial smoothing changes eigenvector centrality values of nodes. Similarly as in the case of degree, the centrality of ROIs located in the temporal lobes increases the most, while eigenvector centrality of most other ROIs decreases. In contrast to Figs. 3 and 4, node and link colors indicate absolute values of eigenvector centrality and link prevalence instead of differences, for networks constructed from non-smoothed and smoothed data. Eigenvector centrality values are averages over 28 subjects.

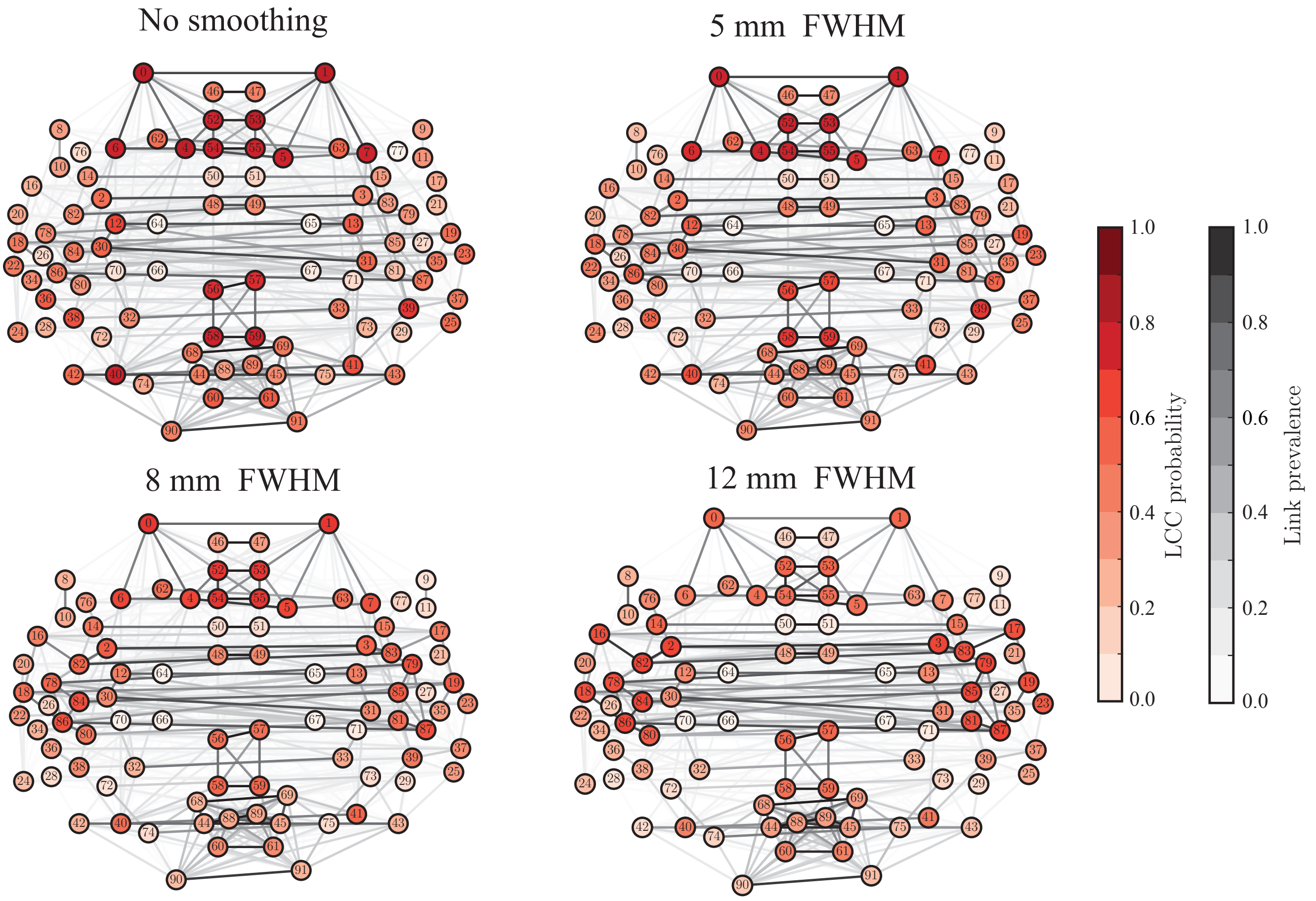

Figure 6: Spatial smoothing changes the structure of the LCC. Although the change is not as clear as in the in-house data, also in the ABIDE data occipital and frontal areas are less probable to belong to the LCC when smoothing has been applied. Node colors indicate how frequently a certain ROI belongs to the LCC in the networks of 28 subjects. Networks are thresholded to 3% density.

3 Supplementary Table

The Supplementary Table is available at https://github.com/onerva-korhonen/effects-of-spatial-smoothing in Excel format (.xlsx). In the Supplementary Table, we present detailed numerical results about the effects of spatial smoothing, separately for each Region of Interest. The table shows names and sizes of the 96 HO ROIs used to analyze the in-house dataset and the 92 ROIs used to analyze the ABIDE dataset, as well as degrees, degree ranks, eigenvector centralities, and probabilities to belong to the LCC calculated with different levels of spatial smoothing applied.

References

Di Martino, A., Yan, C.-G., Li, Q., Denio, E., Castellanos, F. X., Alaerts, K., Anderson, J. S., Assaf, M., Bookheimer, S. Y., Dapretto, M., Deen, B., Delmonte, S., Dinstein, I., Ertl-Wagner, B., Fair, D. A., Gallagher, L., Kennedy, D. P., Keown, C. L., Keysers, C., Lainhart, J. E., Lord, C., Luna, B., Menon, V., Minshew, N., Monk, C. S., Mueller, S., Müller, R.-A., Nebel, M. B., Nigg, J. T., O'Hearn, K., Pelphrey, K. A., Peltier, S. J., Rudie, J. D., Sunaert, S., Thioux, M., Tyszka, J. M., Uddin, L. Q., Verhoeven, J. S., Wenderoth, N., Wiggins, J. L., Mostofsky, S. H., and Milham, M. P. (2014). The autism brain imaging data exchange: towards a large-scale evaluation of the intrinsic brain architecture in autism. *Molecular psychiatry*, 19(6):659–667.

Tyszka, J. M., Kennedy, D. P., Paul, L. K., and Adolphs, R. (2014). Largely typical patterns of resting-state functional connectivity in high-functioning adults with autism. *Cerebral cortex*, 24(7):1894–1905.
