# Supplementary material for: Effects of spatial smoothing on functional brain networks
Source: Eur J Neurosci. 2017 Oct 20;46(9):2471–80. doi: 10.1111/ejn.13717 (PMC5698731; doi:10.1111/ejn.13717)
Supplement: Supplementary file 1 [file EJN-46-2471-s001.pdf]

EJN European Journal of Neuroscience

FENS Federation of European Neuroscience Societies

Effects of spatial smoothing on functional brain networks

Tuomas Alakörkkö, Heini Saarimäki, Enrico Glerean, Jari Saramäki & Onerva Korhonen

Review timeline:

Submission date:	24 April 2017
Editorial Decision:	03 July 2017
Revision received:	22 August 2017
Accepted:	13 September 2017

Editor: Susan Rossell

1st Editorial Decision 03 July 2017

Dear Dr. Korhonen,

Your manuscript was reviewed by external reviewers as well as by the Section Editor, Dr. Susan Rossell, and ourselves.

The reviews collectively indicate that your experiments generated new and important information. However, there are several substantial issues that need to be clarified/resolved before we can consider your manuscript further for publication in EJN.

Overall the paper is written well and addresses an important issue. The last paragraph of the introduction needs rewriting as to only present the aims and hypotheses of the study, and should not include results and interpretations. Both reviewers have indicated that further information is required in regards to the confirmation data set from ABIDE. Were the 28 participants all collected on the same scanner, and what were the scanning parameters and the demographics of the second sample? In section 4.3 you discuss that the findings have implications for diseased participant groups. This statement is a little premature and should either be less definitive or present data using a patient group. Given that you have access to the ABIDE database examining whether spatial smoothing influences the functional brain networks in autism would resolve this criticism of the paper. Although the paper is written well, it is a little repetitive in places, please try to reduce the overall length of the manuscript. Please address the rest of the comments raised by the reviewers.

We also noted the following points.
- Text should be in single column format.
- Section numbering should be removed.
- Footnotes should be removed or relocated to the main text.
- All authors of cited papers should be included in the reference list.
- References are not in EJN style, some journal abbreviations are not correct, some information is missing and all authors of cited papers should be included.

If you are able to respond fully to the points raised, we would be pleased to receive a revision of your paper within 12 weeks.

Thank you for submitting your work to EJN.

Kind regards,

Paul Bolam & John Foxe
co-Editors in Chief, EJN

Reviews:

Reviewer: 1 (Omar Dekhil, University of Louisville, USA)

Comments to the Author

1- the number of subjects used in testing is very limited, there are too many data sets that have resting state fMRI, that include but not limited to national database for autism research (NDAR)
2- There are many previous studies that stated the importance of spatial smoothing on increasing SNR, (for example Triantafyllou et al, 2006 and Mikl et al 2008) , but this study does not compare or criticize there work.
3- In this study, it is claimed that the spatial smoothing may decrease the differences between two studied groups. It would be great to show this by an experiment on two healthy and patient groups and demonstrate the negative affect of spatial smoothing
4- The ABIDE dataset contains more than 500 subjects, why those 28 subjects were chosen?

Reviewer: 2 (Marwa Ismail, Case Western Reserve University, USA)

Comments to the Author
The paper discusses an important topic in brain imaging using fMRI, which is how much spatial smoothing is useful in the preprocessing of fMRI signals. The paper is well written, with a comprehensive literature and a detailed experiment. However, I have difficulty tracking the number of subjects used for the experiments. Is it only the 28 controls mentioned? I see that the ABIDE database, which is a huge one publicly used for Autism, is exploited. Yet, it is not very clear how many subjects are used, or the age group, and the type of fMRI experiment conducted. The ABIDE database includes many different databases collected from different sites, so please try to be specific in providing the details of the subjects the experiment was validated on. Also, why did you only apply the method of smoothing on only controls? It would be interesting to see how your algorithm would perform on signals coming from autistic subjects as well and if it is going to affect or remove important information from BOLD signals of them.

Authors' Response 22 August 2017

Editor:
Overall the paper is written well and addresses an important issue.

We thank the Editor for the positive comments.

The last paragraph of the introduction needs rewriting as to only present the aims and hypotheses of the study, and should not include results and interpretations.

We apologize for not being aware of this style requirement for the introduction; we have now updated this paragraph and removed results and interpretations.

Both reviewers have indicated that further information is required in regards to the confirmation data set from ABIDE. Were the 28 participants all collected on the same scanner, and what were the scanning parameters and the demographics of the second sample?

The ABIDE dataset used in the present study contained 19 subjects measured at California Institute of Technology (Caltech) and 9 subjects measured at Carnegie Mellon University. In order to keep the manuscript concise, the full details of the ABIDE dataset, including the subject IDs and detailed scanning parameters, are described in the Supplementary Methods. Nevertheless, we have also expanded the description of the ABIDE data in the Methods section, and refer the reader to the Supplementary Methods wherever necessary.

In section 4.3 you discuss that the findings have implications for diseased participant groups. This statement is a little premature and should either be less definitive or present data using a patient group. Given that you have access to the ABIDE database examining whether spatial smoothing influences the functional brain networks in autism would resolve this criticism of the paper.

We agree; showing with data that subject groups get increasingly similar would indeed settle the issue. However, this is less straightforward than it initially seems: first, for a conclusive result, several subject-control group pairs should be investigated, and second, more importantly, there is no common way of quantifying network differences that would be applicable to any pair of networks (typically, differences are assessed with measures tailored for each study). Therefore, we have chosen to moderate our claim and we now present it in the form of a question instead; we leave the proposed comparison for future work as it would easily contain material for a paper of its own, if the methodological problem of comparing networks is included. Please also see our response to Reviewer 1 who raised the same question.

Although the paper is written well, it is a little repetitive in places, please try to reduce the overall length of the manuscript.

We thank the editor for the positive comment; we have now cut down the length of the paper and removed repetitive sentences, which has indeed improved the manuscript. In order to not reduce readability of the manuscript, we have not highlighted all these changes.

Reviewer 1:
1- the number of subjects used in testing is very limited, there are too many data sets that have resting state fMRI, that include but not limited to national database for autism research (NDAR)

In our case, we see similar effects of smoothing with two independent datasets: this is in our view sufficient to point out that spatial smoothing may cause unwanted side-effects in network-oriented analysis of fMRI data. We agree that using more data might allow a more detailed analysis of the effects of smoothing, e.g. detecting network edges that always get stronger in all data sets and subjects; however, our aim here has been to show that this effect exists, and for that purpose, we argue that two independent datasets are enough. We have added the following sentence to Discussion: "While increased numbers of subjects or additional data sets might reveal detailed effects of smoothing on particular nodes or links, our results with two sets of data clearly point out that spatial smoothing has unwanted side effects on functional brain networks."

2- There are many previous studies that stated the importance of spatial smoothing on increasing SNR, (for example Triantafyllou et al, 2006 and Mikl et al 2008) , but this study does not compare or criticize there work.

While we already cited Mikl et al, we have added citations to the work of Triantafyllou. In the Introduction it is stated that "Spatial smoothing also increases signal-to-noise ratio (SNR), compensates for inaccuracies in spatial registration, and decreases inter-subject variability (Hopfinger et al., 2000; Triantafyllou et al., 2006; Bennett & Miller, 2010; Mikl et al., 2008; Pajula & Tohka, 2014}. Spatial smoothing is often applied outside the GLM paradigm as well; in this case the justification for using it is less evident." We also discuss SNR in the last paragraph of Discussion, where we have added a citation to Mikl as well.

3- In this study, it is claimed that the spatial smoothing may decrease the differences between two studied groups. It would be great to show this by an experiment on two healthy and patient groups and demonstrate the negative affect of spatial smoothing

We agree with the referee; however, conducting a comprehensive comparison would require several pairs of data sets as well as a common method for quantifying differences between networks which doesn't yet exist. Therefore, we have moderated our claim and present it now in the form of a question as: "The decrease of inter-subject variation raises the question whether variation is also decreased between subject groups that are supposed to be different, e.g., patients and controls. This should be tested in further experiments, where the differences in network structure between two groups are measured with and without spatial smoothing. Unfortunately, a comprehensive comparison is made difficult by the lack of a general way of measuring differences between networks; typically, they are quantified with methods that have been tailored for each study."

4- The ABIDE dataset contains more than 500 subjects, why those 28 subjects were chosen?

We used two criteria for selecting the subjects. First, we wanted to exclude all children younger than 17 years; our inhouse data do not contain such children, and as human brain networks are known to change by age, including the children might have caused differences between the two datasets. Second, as we pooled all the selected ABIDE subjects together in order to form a single dataset, they all needed to have the same repetition time (TR). So, we picked the maximum number of adult subjects with a single TR. In the case of the selected ABIDE subjects, TR was 2.0s, which is relatively close to the TR used to collect our in-house dataset (1.7s). We have added in the Methods section of the manuscript a paragraph describing the reasons for selecting the ABIDE subjects.

Reviewer 2:
The paper discusses an important topic in brain imaging using fMRI, which is how much spatial smoothing is useful in the preprocessing of fMRI signals. The paper is well written, with a comprehensive literature and a detailed experiment. We thank the reviewer for their positive comments. However, I have difficulty tracking the number of subjects used for the experiments. Is it only the 28 controls mentioned? I see that the ABIDE database, which is a huge one publicly used for Autism, is exploited. Yet, it is not very clear how many subjects are used, or the age group, and the type of fMRI experiment conducted. The ABIDE database includes many different databases collected from different sites, so please try to be specific in providing the details of the subjects the experiment was validated on.

We used two independent datasets: 13 subjects measured in-house and 28 subjects from the ABIDE I initiative. In order to avoid extensively increasing the length of the manuscript, the full details of the ABIDE subjects, including the subject IDs and detailed data acquisition parameters, are described in the Supplementary Methods. However, we have also expanded the description of the ABIDE dataset in the Methods section of the main manuscript.

Also, why did you only apply the method of smoothing on only controls? It would be interesting to see how your algorithm would perform on signals coming from autistic subjects as well and if it is going to affect or remove important information from BOLD signals of them.

In this paper, our target has been to show that spatial smoothing induces artefacts in functional network structure. We agree that it would indeed be interesting to see if the effects are any different in e.g. autistic subjects; however, a comprehensive comparison would require developing ways of quantifying network differences (see our responses to the Editor and Reviewer 1) and we feel that this is beyond the scope of the present paper.
